# Sharing and Empathy in Digital Spaces: Qualitative Study of Online Health Forums for Breast Cancer and Motor Neuron Disease (Amyotrophic Lateral Sclerosis)

**DOI:** 10.2196/jmir.9709

**Published:** 2018-06-14

**Authors:** Sarah Hargreaves, Peter A Bath, Suzanne Duffin, Julie Ellis

**Affiliations:** ^1^ Health Informatics Research Group Information School University of Sheffield Sheffield United Kingdom; ^2^ Department of Sociological Studies University of Sheffield Sheffield United Kingdom

**Keywords:** online health forum, sharing, breast cancer, motor neuron disease, amyotrophic lateral sclerosis, empathy

## Abstract

**Background:**

The availability of an increasing number of online health forums has altered the experience of living with a health condition, as more people are now able to connect and support one another. Empathy is an important component of peer-to-peer support, although little is known about how empathy develops and operates within online health forums.

**Objective:**

The aim of this paper is to explore how empathy develops and operates within two online health forums for differing health conditions: breast cancer and motor neuron disease (MND), also known as amyotrophic lateral sclerosis.

**Methods:**

This qualitative study analyzed data from two sources: interviews with forum users and downloaded forum posts. Data were collected from two online health forums provided by UK charities: Breast Cancer Care and the Motor Neurone Disease Association. We analyzed 84 threads from the breast cancer forum and 52 from the MND forum. Threads were purposively sampled to reflect varied experiences (eg, illness stages, topics of conversation, and user characteristics). Semistructured interviews were conducted with 14 Breast Cancer Care forum users and five users of the MND forum. All datasets were analyzed thematically using Braun and Clarke’s six-phase approach and combined to triangulate the analysis.

**Results:**

We found that empathy develops and operates through shared experiences and connections. The development of empathy begins outside the forum with experiences of illness onset and diagnosis, creating emotional and informational needs. Users came to the forum and found their experiences and needs were shared and understood by others, setting the empathetic tone and supportive ethos of the forum. The forum was viewed as both a useful and meaningful space in which they could share experiences, information, and emotions, and receive empathetic support within a supportive and warm atmosphere. Empathy operated through connections formed within this humane space based on similarity, relationships, and shared feelings. Users felt a need to connect to users who they felt were like themselves (eg, people sharing the same specific diagnosis). They formed relationships with other users. They connected based on the emotional understanding of ill health. Within these connections, empathic communication flourished.

**Conclusions:**

Empathy develops and operates within shared experiences and connections, enabled by structural possibilities provided by the forums giving users the opportunity and means to interact within public, restricted, and more private spaces, as well as within groups and in one-to-one exchanges. The atmosphere and feeling of both sites and perceived audiences were important facilitators of empathy, with users sharing a perception of virtual communities of caring and supportive people. Our findings are of value to organizations hosting health forums and to health professionals signposting patients to additional sources of support.

## Introduction

### Background

The availability of an increasing number of online health forums has altered the experience of living with a health condition because more people are now able to connect and support one another [1]. This relatively accessible means of communication is particularly important when someone is living with a health condition that is rare or which inhibits communication (eg, motor neuron disease [MND]) [2]. Online support communities provide various types of support, such as emotional [3-5] and informational support [2]. One aspect of human emotional intelligence, which may, in part, shape online communication within forums, is empathy. Previous research has highlighted the role of empathy in encouraging helpful behaviors, such as motivating users to share knowledge [6], and the potential benefits users derive from empathetic communications [7]. However, less is known about the processes which facilitate social and relational connections, such as empathy, within online forums [1]. The study reported in this paper addressed this by exploring how empathy develops within these spaces, is enacted, and operates within two online health forums—one for individuals living with or affected by breast cancer and another for people living with or affected by MND. Although most research on forums and online communities tends to focus on a single health condition [1,8], we sought to enrich our understanding of empathic processes by exploring the lived experience of individuals engaging with two different online health forums.

### Definitions of Empathy

There are many definitions of empathy and these have been applied inconsistently within research [9]. However, there are aspects of empathy that are commonly discussed, such as *cognitive empathy* (the capacity to understand another person’s feelings) and *affective empathy* (the capacity to respond with appropriate emotion). A less commonly discussed aspect is *congruence* in empathy, which is the requirement for empathetic emotion of the observer to correspond with that of the observed. For the purposes of this paper, we define empathy as “(1) knowing what the other person is feeling, (2) feeling what the other person is feeling, and (3) responding compassionately to another person’s distress” (p 234 [10]). This definition was adopted for the purposes of this paper because it resonated with participants’ understandings of empathy, which had emerged during the interviews. Participants with both health conditions understood empathy chiefly as the ability to understand what another is feeling and to stand in another person’s shoes. Many noted the ease with which they were able to achieve this with forum members because of their shared illness experiences. This enabled them to both (1) know and (2) feel. Participants linked thought with action, requiring empathy to be demonstrated with (3) a compassionate response. Within previous studies on online health forums, focusing on a variety of topics, there are elements within the findings that correspond with this definition:

1. Knowing what another person is feeling, such as users with shared very specific understandings of living with particular conditions (eg, [1,11,12]).
2. Feeling what another person is feeling, such as users recognizing emotions and feeling a resonance (eg, [13]).
3. Responding compassionately to another person’s distress, such as written responses to problems expressed on the forum, demonstrating compassion by offering mutual understanding [14], sharing personal experience [14], validating feelings [8,15], or responding promptly to requests for support [14,16].

This paper, therefore, makes a new contribution to understanding of empathy in eHealth in that it explores the importance of these three aspects of empathy for people with life-threatening conditions using online health forums and their communication online. In the following section, we outline previous research that has examined the role of empathy in online communication and in online health forums.

### Empathic Communication Online

For online health forums to flourish, users must be prepared to share knowledge and experiences with others in the community [6] and to develop trust in how the forum operates and with others who use it [17]. Empathy is thought to encourage and motivate sharing [6], and thus has been studied by researchers seeking both to understand the mechanisms that facilitate a forum’s usefulness and to consider how software might facilitate empathetic communication [18]. To date, research on empathy has focused on assessing whether empathy is present within messages posted on online communities [19] and on how empathy is expressed and structured within interactions among users [19,20]. Studies have examined differing levels of support provided by users to group members in need, with empathic exchanges thought to offer a greater depth of support [19,21]. The emotional depth conveyed through empathic interactions emanates from an understanding gained from others having shared their experiences of being in a similar situation and the ability to imagine and work oneself into the emotions and situation of others [21]. Empathetic support requires effort, understanding, and caring [19].

Studies have identified the factors that may increase the likelihood of empathetic exchanges or strengthen empathetic bonds. Empathy is stronger or more evident when users share similarities, such as going through the same experience. Similarity provides a basis for greater identification between users, increases the likelihood of an accurate understanding of the situation (empathetic accuracy), and fosters greater intimacy [12,14,19,22]. Gender also influences these processes, with more empathy present in communities with a female membership [23]. Communications among women tend to have greater empathic content and lower factual content, and communications between men typically have lower empathic content and higher factual content [18]. Age is also a factor in influencing emotional and empathetic communication online, with one study showing that teenagers send more emotional messages in a personal style, in contrast to the formal style of older adults [24]. However, older adults within this study expressed higher levels of concern for others than their teenage counterparts. Crucially, empathy is influenced greatly by the topic of interest, and is found to be most present within support communities [25,26]. Messages conveying emotions and feelings are the most common trigger for an empathic response [19,20]. Empathy is also encouraged by the altruistic sharing behaviors of users, such as sharing personal knowledge, feelings, and experiences [6], and moving the focus from self to offering peer support [13]. Empathy is commonly expressed in responses to requests for support [27]. Self-disclosure is an important element of online communication [19], which informs empathic interactions among users.

### Empathy and Online Health Forums

Studies focusing on online health forums have explored the context in which online interactions take place as users work through illness experiences, interact with health services, and experience the impacts of ill health on offline relationships and everyday lives [1,8]. This context influences empathy building within health forums as users live with the ongoing stresses and challenges of ill health. For people living with health conditions that may or will shorten their life, the need for empathetic support and understanding can be particularly important [28,29]. Shared understanding and experiences of living with ill health found within condition-specific health forums have been shown to help foster a sense of community and build friendships [1]. These factors encourage empathetic behaviors because users seek to nurture and support one another [30]. However, this relatively limited research on empathy in online health forums has not considered the processes by which empathy is developed in online health forums and this study sought to fill this gap.

### Study Aims

The aim of our study was to develop a better understanding of the role of empathy among people who share information, experiences, and emotions in online health forums. More specifically, we were interested in exploring the processes that underpin the development of empathy and influence sharing among users of two online health forums for two very different conditions. In this paper, we explore how empathy is developed through shared experiences and via connections formed between users. We used forum data to explore how empathy is narrated within the forums and the interviews to explore, and gain a deeper understanding of, the human processes behind it.

## Methods

### Study Design

This study is part of a wider project exploring how and why people living in a range of extreme circumstances share information, emotions, and experience in online spaces and communities. The key focus was on understanding the role of trust and empathy in encouraging and shaping sharing behaviors. The study recently reported on the processes by which trust develops on a breast cancer forum [17], demonstrating how it operates within structural, relational, and temporal dimensions. This paper explores the development of empathy through sharing in online environments.

The paper focuses on the experiences of people living with two very different health conditions: breast cancer and MND, also known as amyotrophic lateral sclerosis. Breast cancer is both a life-threatening and a long-term condition given that many people survive for 10 years after diagnosis [31], whereas others may die of it or associated conditions. It is not a single condition; there are different stages and types of cancer, with differing prognoses, treatment options, and choices. People with breast cancer face differing trajectories and, for some, the condition can be terminal. In contrast, MND is a relatively rare and incurable condition, and people diagnosed with one of the diseases within this group can expect to have their life severely shortened [32]. MND causes damage to nerves and muscles, and as the disease progresses people may experience difficulties with mobility, speech, eating, drinking, and breathing. The lack of a curative treatment means that people face an inevitable decline. These two health conditions were chosen to explore aspects of living in extreme circumstances with two quite different conditions: one that is potentially life threatening (breast cancer) and the other which is life shortening and for which there is no cure (MND).

We explored how empathy develops within two online health forums that provide support for people living with breast cancer and with MND. We utilized a case-study approach to gain an in-depth understanding of the phenomenon, undertaking qualitative analysis of a sample of threads from the online forum provided by Breast Cancer Care (a UK-based charitable organization) and a forum hosted by the Motor Neurone Disease Association (a charity providing support in England, Wales, and Northern Ireland). We also conducted semistructured interviews with 20 users of these forums. The combined datasets broadened our understanding of the issues raised because each source illuminated particular aspects of the forum experience and additional insights were gained from analyzing connections between the threads and interview material. Although analysis of combined data sources is not commonly undertaken [8], in this instance, it provided the opportunity to triangulate analyses and reach a better understanding of how empathy develops in the forums [17]. The forum posts demonstrated how empathy is presented within postings and the interviews provided insights on participants’ thoughts and feelings about empathy within the forums, and how this informed their interactions and sharing practices within these online spaces.

### Study Setting

We approached and gained permission from two leading UK charities to access and analyze forum posts and to invite forum users to participate as interviewees in the study: Breast Cancer Care and the Motor Neurone Disease Association. These particular forums were chosen for a number of reasons. Both forums met the inclusion criteria, they are open access (in that anyone may view and read the messages without having a user ID and password), and the terms and conditions stated that it is permissible to use the forum data for research purposes. The two forums were also chosen to explore differing research settings. The two forums differ in the following ways:

1. Size of membership: Breast Cancer Care is a relatively large forum—at the time of the study, it was estimated there were 200,000 registered users (Breast Cancer Care, personal communication). MND is a smaller forum, with more than 3000 registered users, although approximately 50 to 100 of these are active users (MND, personal communication).
2. Length of time established: Breast Cancer Care is one of the longest established Web-based forums; it was established in 1999-2000. The MND forum is more recent; it was established in 2013-2014.
3. The health conditions are distinctive and different (as previously described).
4. Gender differences: the Breast Cancer Care forum is predominantly female and the active members of the MND forum are predominantly male.

Both sites are moderated by staff employed by the charities. Both forums were structured to reflect both stages within user journeys (eg, diagnosis), user characteristics (eg, carers), and aspects of living with the condition. The Breast Cancer Care forum provides a wider range of boards in which to share experiences, reflecting journeys across differing trajectories.

### Forum Threads

The researcher (JE) spent time reading through the posts to familiarize herself with both sites and gain an understanding of the context and feel of the online environment and characteristics of the differing boards [17]. We undertook a two-stage process to download a sample of forum threads from the two forums. In the first stage, we purposively selected discussion boards from the two forums to provide suitable diversity of the topics discussed. In 2015, 233 threads were downloaded from 10 boards on the Breast Cancer Care forum and 135 were downloaded from five boards on the MND forum. The threads spanned a range of years (2006-2014), reflecting the different dates in which the boards originated. The posts were collected from the start date of the board until the sample quota was reached. The quota was decided pragmatically, the calculation based on the volume of threads within each site, and a requirement to ensure that the amount of data downloaded was manageable. The data collection strategy was designed to ensure that the forum threads sampled were collected in a consistent way across the differing boards. From these, a subset of 84 threads from the Breast Cancer Care forum and 52 from the MND forum were purposively selected to reflect varied experiences (eg, illness stages, topics of conversation, and user characteristics; Table 1). Sampling was undertaken with an awareness of the study purpose to explore concepts of empathy, and a wider focus on sharing and trust. The broad sampling strategy sought to ensure the study gained an understanding of both how empathy develops and operates; therefore, we included threads in which empathy was very apparent (eg, emotional threads, moments of need), threads in which empathy could be perceived as lacking (eg, moments of conflict), and threads in which empathy was not apparent or immediately apparent (eg, everyday conversations).

**Table 1:** Details of forum posts included in the analysis.

Forum type and section		Board	Date of posts	Threads analyzed, n
**Breast Cancer Care forum**				
	Talk to people like me	Men’s board	2006	10
	Talk to people like me	Younger women and families	2007	10
	Welcome to the forum	New members board	2012-2013	12
	Going through treatment	Surgery	2007	11
	Going through treatment	Chemotherapy (monthly)	2013	1^a^
	I am recently diagnosed	Diagnosed with breast cancer	2007	10
	Have I got breast cancer?	Appointments and waiting	2007-2008	10
	I have secondary breast cancer	End-of-life board	2009-2010	10
	Living with and beyond breast cancer	Coping with fear and anxiety	2012-2014	5
	Living with and beyond breast cancer	Sex and relationships	2012-2014	5
**Motor neuron disease forum**				
	Help and advice	Tips and experiences	2011-2013	10
	Miscellaneous discussion	Off topic	2011 and 2013	10
	General discussion	Life with MND	2010-2013	11
	General discussion	Introduce yourself	2010-2013	11
	General discussion	For carers	2010-2013	10

^a^The chemotherapy board differed from other boards because it was in one long thread. Stages within one thread were sampled (eg, start, one-quarter way through, halfway through, and so on).

### Interviews

The interview participants were recruited by posting a message on the Breast Cancer Care and Motor Neurone Disease Association forums. The message explained the aim of the study and people who were interested in participating were invited to contact the study team. Criteria for interview were as follows: participants were aged 18 years and older, users of the forums with a diagnosis of breast cancer or MND, or a relative or friend of someone with the condition. All interview volunteers recruited were people living with the health conditions, with the exception of the partners of two participants with MND who sat in on and contributed to the interviews. Interviews were conducted by JE either face-to-face, via phone, Skype, or email. The final sample, therefore, was self-selecting (ie, those people who contacted the study team to take part). Although there is potential for a biased sample, this method yielded a range of experiences (Table 2). Some interview participants with MND communicated via a speech synthesizer in face-to-face sessions and one participated via email. Interviews were conducted at a time and in a place convenient for the interviewees, typically at home. Table 2 shows the characteristics of the interviewees. There were 14 breast cancer interviewees and five MND interviewees.

The interviews were semistructured, and a topic guide was used to prompt discussion on topics relating to the person’s use of the forum, their experiences of sharing online, relationships with other forum users, and experiences and perceptions of how trust and empathy operated in the forums. A flexible schedule was used to guide participants to issues of relevance to the study, but also to give freedom to explore other aspects of importance to participants. The topic guide included asking participants about their use of the Internet and online health forums; their experiences of online sharing of information, resources, stories, emotions, and experiences; their relationships with other people they met in the forums; and how they experienced trust and empathy in relation to other forum users. The duration of the breast cancer interviews ranged from 49 minutes to 2.5 hours, with an average length of approximately 1 hour. The duration of the MND interviews ranged from 1.3 hours to 2.4 hours, with an average length of 1.7 hours; this longer duration reflected the slower pace of communication for people with MND. All interviews were audio-recorded and fully transcribed. After each interview, field notes were taken by JE to document any immediate contextual and analytical insights.

### Data Analysis

The data were analyzed thematically following the methodology outlined by Braun and Clarke [33]. In the first stage (familiarization) two researchers (SD and SH) read through the datasets (forum posts, interview transcripts, and field notes). SH took the lead on analysis for this paper and, in the next stage, discussed initial ideas with the interviewer (JE) and the lead investigator (PB). Initial coding was undertaken manually to give a greater immediacy to the data sources. Comparisons were made with earlier initial coding developed by JE. A subset of data were coded independently by SH and SD to check consistency of coding and data interpretation. The data were then analyzed thematically by SH using NVivo 10 software, with interpretation of codes discussed with JE, PB, and SD. Codes were grouped into themes and then reviewed and refined by rereading data extracts, thus ensuring a fit between the datasets and interpretation. Data interpretation was discussed in an ongoing dialog between SH and SD to further ensure that analysis reflected participant experience. Findings were also discussed with PB. The analysis was also informed by interactions with members of the public at dissemination events for the study, and who shared experiences of online health forums.

Data analysis from the interviews yielded a rich understanding of the role of empathy in online communication and in these online health forums, with a variety of perspectives (generating a breadth of understanding) and a detailed understanding of themes (depth of understanding). It was unnecessary to seek further interviewees.

### Analysis of Both Data Types

The datasets (interview and forum posts) were analyzed separately and then together to gain a better understanding of how empathy operates within the forums. This interplay between datasets enabled the study to explore a greater range and depth of understanding because each data source revealed both overlapping and differing aspects.

We did not seek to understand empathy at a fixed point in time. The data sources were not anchored to one single time point. Interviews and forum samples covered a range of time frames, which did not necessarily overlap. However, this was not considered inconsistent given that memories and experience bridge a wide time frame. This approach was considered appropriate to gain a broad understanding of how empathy operates within the forums. These varied time frames do not preclude triangulation of data sources because it provided a means of exploring consistency of themes across the differing sources and times.

Themes were informed by both sources. The theme “transformative experience,” for example, came initially from interview data where interviewees from both forums gave vivid accounts of life-changing experiences of illness onset and diagnosis. These events happened “off-stage” from the forum. The interview data provided a depth of understanding of the devastation caused by diagnosis, and the process by which this both created a need for empathetic understanding and drew interviewees to the forum, where they could share their story with others in the same boat. These events were often summarized in introductory posts on the forums.

**Table 2:** Participant characteristics (N=19).

Forum and participant (pseudonym)		Age range of person with illness		Gender		Interviewee		Length of time since diagnosis		Period/time of joining the forum		Current level of activity on the forum	
**Breast Cancer Care forum**													
	Anne		50-59		Female		User		~15 months		Joined before formal diagnosis		Posting daily and checking posts
	Beth		40-49		Female		User		3 years		At biopsy		Less frequent contact with forum; moved onto Facebook group with friends
	Christine		50-59		Female		User		~13 months		1 year ago (joined chemo thread)		Checking posts and posting daily
	Danielle		40-49		Female		User		~4 months		~4 months ago		Checking post every 3-4 days
	Eleanor		50-59		Female		User		~2.5 years		2 years ago		Checking posts 2-3 times a week
	Frances		50-59		Female		User		~16 months		Started using the forum at chemo		Checking posts twice weekly
	Gayle		50-59		Female		User		~18 months		Started using the forum at chemo		Checks posts once or twice a week
	Hazel		60-69		Female		User		~3 years		Joined ~19 months ago		Accessing forum at points of worry
	Isobel		40-49		Female		User		~15 months		Joined chemo group ~15 months ago		Regularly checks posts
	Janice		60-69		Female		User		~13.5 months		Joined chemo group (~13 months ago)		Regularly checks posts
	Kathryn		50-59		Female		User		First diagnosis: 23 years; second diagnosis: 4.5 months		~4 months ago		Checks posts daily
	Libby		40-49		Female		User		~10 months		Since chemo (~8 months ago)		Checks posts twice a day
	Nancy		40-49		Female		User		~7 months		Since diagnosis (~7 months ago)		Lurker (reading posts twice daily)
	Olivia		50-59		Female		User		~5 years		Since diagnosis (~5 years ago)		Checks forum every 6-8 weeks
**Motor neuron disease forum**													
	Pippa & Michael		60-69		Female		User and partner		10 months		First went on forum 3 months before formal diagnosis		Daily user
	Robert & Meg		60-69		Male		User and partner		~19 months		Joined forum at unidentified point and become active user 3 months ago when realized the disease was becoming more aggressive		Daily user
	Sue		70-79		Female		User		5 years		Joined forum 4.5 years ago		Daily user
	Terry		70-79		Male		User		~4 months		Joined forum soon after diagnosis		On the forum most days
	Vincent		60-69		Male		User		~3 years		Forum member for 3 years		Very active—checking posts 2-3 times a day

### Ethical Considerations

Both forums are openly accessible by anyone choosing to read the message boards online, although users are required to register and log in to actually make posts. At the time of the study, terms and conditions in each forum stated that posts were publicly visible and, with permission of the charitable organization, the posts may be used for research purposes. Recommendations provided by the University of Sheffield’s Research Ethics Committee regarding the forum data were followed, with steps taken to preserve user anonymity by removing individual identifiers and changing details that might identify individuals or organizations. When preparing material for publication, we have reworded forum posts carefully to retain their original meaning and nuance while ensuring that phrases cannot be used in Internet search engines to trace quotations back to individual users.

Potential interviewees were provided with study information sheets and a copy of the consent form at least 24 hours prior to the interview to obtain informed consent. Interviewees were informed that they could stop the interview and/or withdraw from the study at any time up until the point of publication. We were mindful that participants may become distressed during the interview, and thus a protocol was devised with both charities to manage this sensitively and ensure, when necessary, participants were appropriately signposted to support. To prepare for interviews with individuals with MND who may experience communication difficulties, JE spent time discussing appropriate approaches with the Motor Neurone Disease Association.

Ethical approval was granted by the University of Sheffield Research Ethics Committee (analysis of forum posts: application 001955) and UK Ministry of Defence Research Ethics Committee (interviews: application 614/MODREC/14).

## Results

Through our analysis, we developed a conceptual framework representing how empathy developed and operated within the forums. We found that empathy was built on shared experiences and connections (Textbox 1). In this paper, we explore these themes, shared experience, and connections, and also discuss their subthemes to highlight how empathy develops in online health forums. An additional theme—knowledge—will be discussed in further work.

### Empathy Built on a Shared Experience

Users of the forums had one thing in common: that in some way their lives had been affected by the diagnosis of a life-threatening health condition. The majority of users were individuals who had been diagnosed with the health condition, although some experienced this indirectly as family caregivers. This bond of a shared experience and, most importantly, of knowing what it felt like to receive a diagnosis and live with a serious health condition, formed the shared emotional backdrop and common understanding within both forums.

Across each forum, users shared some similar experiences and emotions relating to diagnosis, but the process from becoming aware that they had a serious illness to diagnosis was quite different for the two conditions. Users of the Breast Cancer Care forum typically described a speedy transition to the world of ill health, going from the assumption that their breast lump would be benign, to receiving a diagnosis that was often both unexpected and shocking, to then often having to adapt swiftly to treatment regimens:

...the shock of the diagnosis, the fact that the treatment was starting so quickly, the operation and all of that, um, and it was kind of just trying to [pause] internalize it all and make sense of it and deal with all the different emotions and kind of my kids and my husband and everything...

Christine, breast cancer interviewee

The process of diagnosis was typically slower for MND participants: there was an awareness of things going wrong with their body and then the uncertainty of undergoing diagnostic tests before a diagnosis was eventually made. However, receiving the diagnosis was still a devastating blow and, for some participants, the hopelessness of their situation was made worse by the way the diagnosis was communicated:

...and the neurologist said to me...in a very blunt fashion...“I think it’s MND.” I’ll make a second opinion and I asked, “What’s MND?” Because I hadn’t a clue. Um, he said, “Well, let me say you’d best go home and sort your affairs out and do what you want to do because,” he said, “two to five years.”

Vincent, MND interviewee

For both users with breast cancer and MND, the transition to ill health and diagnosis was a traumatic experience as they sought to deal with the emotional impact and to live with uncertainty. At this point of need, most participants found the forums by chance by searching for sources of help on the Internet. Only two interviewees in our study, one with breast cancer and one with MND, were directed to the forums by a health professional.

Introductory posts on both forums summarized people’s experiences, offering an important starting point for forum users to reach out and connect:

Thank you everyone for sharing all of that. Everyone’s experiences are really important so other people can empathize with...

moderator post, MND forum, in response to someone’s post

It’s Saturday 16th June & I was diagnosed (grade 3) last Wednesday. The tumor is 1.5 cm, which sounds not too bad to me. I’m booked in to have a [name of procedure] op next Weds. At the moment, it’s difficult to come to terms with the speed of it all, and I feel as though I am totally in limbo. Just waiting for Wednesday really.

breast cancer forum post

**Thematic map of themes and subthemes for how empathy is built with the breast cancer and MND forums.**

Theme 1: Shared Experience

1. Transformative experience

Theme 2: Connections

1. Forum community
  - A meaningful space
  - Feeling set apart from others
2. Similarity
  - Different but similar
  - Drawn to and seeking out similarity
  - New spaces for empathy
3. Relationships
  - Building relationships
  - Friendship
4. Feelings
  - Expression of feelings
  - Emotional impacts

These quotations demonstrate the need for individuals to share their experiences and emotions with other people at an early stage in their diagnosis. It also gave fellow users the opportunity to provide empathic responses. This information sharing was fundamental to the process of building connections between forum users and provided a basis on which to express, offer, and experience empathy.

### Empathy Built on Connections

The second theme, “connections,” explores the means by which empathy is built through differing types of connections that are developed within the forums. The first aspect of this was having a sense of being connected to online support communities (eg, within the Breast Cancer Care or Motor Neurone Disease Association forums), as well as the wider community of people living with these conditions:

I do see it as a supportive community...

Terry, MND interviewee

It is important to remember is that we will all be helped and supported through our distressing experience by the others on this site who have or are “walking in our shoes”—and for which I have found a godsend!

breast cancer forum post

Within this theme, we explore key attributes of the forum that drew interviewees to the forums and enabled them to form an attachment to the people within these spaces.

#### Connected by Forum Community

##### Finding a Meaningful Space

Interviewees with both health conditions perceived the forums as meaningful spaces where they could share their own experiences and emotions with people in a similar position and receive an empathic response:

I’ve certainly found it useful myself, certainly to know that there’s people there who really understand what you’re going through, your thoughts and worries, et cetera.

Anne, breast cancer interviewee

You need to be around people who are rowing in the same boat trying to keep afloat in the face of real adversity and on the forum you find this empathy because it is so unique. There is nowhere else like it.

Pippa, MND interviewee

There was also recognition that the forums could be a unique source of information as well as an emotional support, provided by others undergoing similar journeys and who shared their own experiences and information:

[The forum is] a safety net of, you know, good, solid information and the most enormous amount of kind of warmth and support from other women, yeah.

Olivia, breast cancer interviewee

We all have a real need for this forum as a platform for our fears and anxieties, for advice, for guidance, for solace, and for friendship.

Pippa, MND interviewee

All the interviewees perceived this support to be useful and relevant to them, and it was also considered beneficial in helping them through difficult situations. Both forums were perceived as supportive and welcoming spaces, offering warmth, comfort, and human understanding. These qualities made the spaces conducive to empathy, as shown in Table 3.

**Table 3:** Qualities that made the communities conducive to empathy (perceptions derived from interviews with people with the health conditions).

Elements	Breast Cancer Care forum	Motor Neurone Disease Association Forum
Support	Source of information (a repository), expertise, help, advice, emotional support, saying what I needed to hear, a means of survival	Help, advice, assistance, finding the answers, friendship, usefulness, support (a support group), a lifeline
Community spirit	Comforting, welcoming, warmth, human contact, checking up on people	Warmth, caring, peaceful, sensitivity, give one another a cuddle, hope
A space to go to	A safety net, picking people up when they are down, a place to go in dark moments, sharing problems	A space to share, an outlet
Tone	Noncritical, nonthreatening, supportive, nonjudgmental, respectful, positive	Nice people, core people, jokes and banter, positive, respectful, but at times it could be contentious
Understanding	Unique understanding, understanding that mental well-being is important	A unique understanding, sharing the same boat

These humane qualities were especially appreciated by participants (particularly those with MND) who had felt this quality lacking from some of their interactions with health professionals. Human contact and connection was a key factor in the sense of attachment that participants felt toward the forums. Although they were connecting to an online space where they could not see others within the forums, there was a common perception that they were joining a group of people sharing the same situation:

...it’s kind of human contact with people in a similar situation.

Gayle, breast cancer interviewee

I just thought there was a lot of support on it [the MND forum] from people who were in the same boat.

Pippa, MND interviewee

##### Feeling Set Apart From Others

Interviewees from both health conditions reported that ill health distanced them from friends and family, who had not been through the same transformative experiences. This was also mentioned in posts on both forums:

The whole breast cancer DX [diagnosis] turns life upside down and sadly, some people (including family) just don’t get the impact it can have on the mind and body.

breast cancer forum post

...I’ve got...family support. I’ve got friends’ support. Um, I’ve got...medical support,...but this [the forum] is another form of support because none of the aforementioned people have got this disease, that’s it. That’s the difference.

Terry, MND interviewee

This contrasted with views about members of the forum communities. Interviewees perceived that forums users understood what they were going through because they all had lived through, and therefore shared, these transformative experiences. This enabled participants to share and express concerns, experiences, and feelings with other forum users that could not necessarily be said to family and friends for fear they would not be understood in the same way, and to express things that could only be said to others sharing that same situation:

The amazing thing about talking to fellow breast cancer sufferers is that they totally understand what you’re talking about, no matter how much you think you’re not making sense!

breast cancer forum post

...you know, you feel an affinity with the other people on the forum and you know they understand what it is that’s happening to me...

Vincent, MND interviewee

Some interviewees talked about sharing experiences with forum users with an emotional honesty that they were unable to extend to conversations with family and friends. These honest exchanges were also encouraged by being able to write and share their feelings within an anonymous space. Some participants felt constrained in sharing feelings with family members because they were striving to keep their feelings in check to present a positive face to others close to them who they wanted to protect. Participants from both forums valued the opportunity to offload feelings and fears in forum posts—to express what could not be said to family and friends for fear of burdening them:

I think without the forum I would have had huge depression here because there’s nobody for me to share anything with and I can’t bring my husband down all the time. He doesn’t understand everything anyway...

Christine, breast cancer interviewee

I don’t feel comfortable with talking to [my family] about what the future holds for me...I’m more prepared to share my feelings and fears with the forum members than my immediate family, just because it—I know it upsets them more than it does me.

Vincent, MND interviewee

The interview participants indicated the importance of connecting with a supportive community of others who share similar experiences; they understood very well the emotional impact of living with a serious illness and demonstrated a willingness to share information about their own experiences, which were of value to others. This depth of understanding and reciprocity of emotional exchange opened up opportunities for empathic discourse. Our data suggest that empathic qualities are enhanced further by the atmosphere and ethos of the online space, with users of both forums clearly valuing the comforting and humane qualities they found within the forums. In addition to this, some users experienced a greater depth of empathic connection when they formed connections with individuals or groups of users with whom they shared similar circumstances.

#### Connected by Similarity

Participants from both forums frequently reported feeling a connection to users with whom they felt a shared similarity. These connections operated on different levels, from a fundamental understanding of sharing the same general diagnosis, to an even greater mirroring of experiences, such as sharing a specific breast cancer diagnosis or experiencing similar symptoms and/or rates of deterioration due to MND. This influenced empathic communication, as users reported that they felt better able to converse with those they perceived to be similar to them. This connection stimulated conversation, users were more prepared to invest time in conversing with similar users, and this increased the potential for conversations to develop, increasing the likelihood of empathetic exchanges.

##### Different but Similar

Although people within the forums shared the same broad diagnosis, there were many individual differences in terms of experiences of living with a health condition (eg, varied pace of deterioration in MND, different stages, and types of breast cancer), the treatment and services received, family circumstances, and attitudes:

...it just shows, although all of us may have cancer the similarities end there. Our diagnoses are different and how we respond to treatment is different, and our opinions are all different, let’s welcome that.

breast cancer forum post

...everybody is so different, even if they have the same diagnosis...

MND forum post

Although people were aware that these differences existed, the connections went beyond these, operating at a more profound level:

I don’t know. It’s just you—when you related to people on such a sort of deep and personal and painful part of your life, you just—it just makes a connection, um, that goes beyond the sort of superficial really.

Janice, breast cancer interviewee

Really deep connections because there is no hope, no cure. We are all fighting the same fight with the same enemy.

Pippa, MND interviewee

The quotations indicate that although users were aware of individual differences, these were overridden by the shared experience of living in extremis and often users formed deep connections. However, it was also common for interviewees to talk about strong bonds forged through greater similarities.

##### Drawn to and Seeking Out Similarity

Within both forums, people sought or made connections based on similarity. These similarities varied, such as living in a close geographical location, shared hobbies, past history, family circumstances (eg, children of the same age), or shared attitudes. Users either identified similarities while reading posts and, from this shared similarity, conversations grew, which could potentially lead to friendships. Other connections were sought, with users asking specifically if others shared their experience or situation, or accessing spaces set aside for users with the sought after characteristic. This was evidenced by interviewee data in which participants talked about wishing to connect with others who were very similar to them. Within the MND forum, connections were sought based on shared problems or the pace of their deterioration, and these provided the necessary bridge between people often living without face-to-face contact with others in the same situation:

There have been a few who seem on a similar journey so they make a lasting impact and one I regularly contact by PM

Sue, MND interviewee

It was apparent that some Breast Cancer Care forum users may seek specific connections, posting detailed descriptions of their situation and diagnosis in introductory posts, in order to find users sharing similar circumstances. This was confirmed by interview data, with participants expressing a need to connect with similar others. Moreover, the Breast Cancer Care forum created particular demarcated spaces to connect people sharing specific circumstances or situations. One powerful example of this was the monthly chemotherapy threads that were set up specifically for users (overwhelmingly women) who were commencing chemotherapy within a specific calendar month:

[We were] all going through chemo at the same time. So you were able to talk about symptoms and how you were feeling and that kind of thing...

Isobel, breast cancer interviewee

This provided a context-specific exchange of empathy that was based on temporality. The fact that these particular forum users were experiencing the same kind of treatment at the same time gave them a synchronized experiential basis on which to develop very strong relationships and form subgroups within the forum environment.

##### New Spaces for Empathy

Users could create their own spaces within the forums by requesting a new space within the forum (eg, a themed board, starting a new thread, exchanging private messages, or moving conversations out of the forum and setting up their own closed Facebook groups). This desire for new spaces came from both a perception that people within the wider forum would not necessarily share or understand the same concerns, and simultaneously a need to connect with others sharing the same particular situation, concerns, approaches, etc. In the following quotation, Olivia described how, as a lesbian woman going through breast cancer, she felt a need for a separate space:

...well actually, there are things that people don’t quite get [within the wider forum] and it would be, you know, really nice to have our own forum. So Evie and I between us requested, bullied, pushed, battered Breast Cancer Care until they did put a little bit up [a space for us]...

Olivia, breast cancer interviewee

Olivia had felt an initial reluctance to mention her sexuality within the wider forum, and her first post had felt like “coming out.” For Olivia, this new space meant that lesbian and bisexual women coming to the forum would feel both welcomed and accepted by others who could perhaps more readily empathize with their experiences more broadly. The space encouraged conversations of particular relevance to this group so they could be had without the need to consider reactions from the wider community. This lack of constraint opened up empathetic communications with others sharing a similar experience, creating a space within the online space of the forum.

Users also set up Facebook groups; this appeared to be more common for breast cancer participants. There were many breast cancer Facebook groups. Users set up groups to talk about experiences that were unique to them, such as a specific cancer diagnoses. For Isobel, the opportunity to connect with others sharing the same diagnosis provided a space where she was better able to open up and share with others who she considered could have greater empathy for her situation:

I think it was mainly because they’d all gone through the same cancer I had and they’d all gone through very similar treatments to what I had and they were dealing with very similar fears to what I was...I think for me it was just...the kind of emotional...you know, fear of recurrence or whatever was easier to discuss with the people who were facing very similar situations to me rather than in a wider group...

Isobel, breast cancer interviewee

Users sought to connect newcomers to these spaces, by signposting to the appropriate group. This signposting role was an important means of building empathy by enabling users with a particular situation/characteristic to connect with similar others.

Invitations posted to the forums about joining Facebook groups emphasized certain advantages offered by these alterative spaces—primarily that Facebook was private and the closed groups offered a secure space to share as the messages posted could not be read by anyone who was not a registered member of the group:

...Josie has set up a FB [Facebook] group and many of us in the December group are on it so no other friends or family can go on it...

breast cancer forum post

Being away from the forum allowed forum users to be less anonymous with others who joined the Facebook group; the Facebook groups were not moderated by staff from the forum organization and personal details could be exchanged. However, although some breast cancer interviewees felt that Facebook offered a space to share with greater openness, there was also awareness that closed Facebook groups excluded some users and took valuable conversations away from the forum.

Finally, within both forums, personal messages sent between individual users via direct messaging and email offered an important means of building more personal communications. Users sent messages directly to others whom they perceived needed greater support, giving them the option of further contact via direct messaging or email. Frequently within both sites, this offer was made without obligation, giving the user the choice to make contact only if they wanted. Personal messages were used within both forums to talk about specific issues, which could not be shared openly within the forum, and to expand and discuss issues at greater length. Personal messaging was also used to express concern. If users were absent from the forum, or were known to be going through a difficult time, members would send a personal message checking up on that person and offering support:

...sometimes one of the members will have gone quiet for a week or so or will have posted something that says, you know, we’re feeling really low about something then you don’t hear from them for a day or two. So I’ll just send a little message to say are you alright, you know, do you want to talk? I’m here

Anne, breast cancer interviewee

I messaged her on FB (Facebook)...Getting worried now...hope gets back soon. I miss her.

Hi Rosie, I have also pmd [private messaged] her xx

MND forum posts

These more individual messages differed from those shared on the open forum by their personal nature, focused as they were on offering particular support to one person. The technologies provided a private space in which friendships grew, and where intimate and personal conversations took place.

#### Connected by Relationships

Empathy is a relational experience and it was evident that it was something that developed through forum relationships. Although some users preferred a matter-of-fact relationship with the forum using it as a source of help rather than friendship, the majority of interviewees with both health conditions felt a strong bond to people within the sites. This is not to say that those with a more pragmatic involvement did not feel empathy for other users; however, relationships encouraged a greater depth of empathetic connection.

##### Building Relationships

Over time, relationships developed in both forums. Users connected with one another as described previously, and friendships grew as they shared information about their situation and lives. Participants were drawn to people with whom they felt a particular connection or affinity. Interviewees described “gelling” or “connecting with” others: one breast cancer interviewee described feeling drawn like a “magnet” to her online friend. In the monthly breast cancer chemotherapy groups, initial sharing focused on treatment experiences and the quickly developed to encompass chatting about a whole range of life experiences. A willingness to share more holistically became “culture” for the groups and increased opportunities of dialog between those participating as well as creating more scope to demonstrate empathy (eg, remembering to ask how a holiday was, whether that glass of wine was enjoyed). During early days of treatment, users typically corresponded daily with the group, and with time and sharing, users came to know one another as friends. These friendships could be intense because they were founded at a time of extremis, and thus there was a depth to the relationships that facilitated sharing and empathy:

I suppose it started off with a—bit all about treatment and cancer and all the rest of it but a lot of the time it isn’t now and—and we’ve also been through a lot. Individuals have shared lots of things on the group, things that have happened to them. I mean...one other woman lost her father, another one whose father was extremely ill...you get to know things about people that you would have to be really—you’d have to have quite a sort of established friendship with other people to get to that point but because you go in on this deep level, it’s sort of much easier to talk about those things and share them.

Janice, breast cancer interviewee

This same process of building friendships through sharing experiences also occurred on the MND forum. Users built up a sense of the people they were communicating with through sharing, gaining a sense of their personality, and their inner thoughts:

...she gets to know the people, gets to know their personality, and their thoughts and their fears

Michael, husband of Pippa MND interviewee

Two of the MND participants spent a great deal of time communicating with their forum friends, and this constancy strengthened bonds and deepened relationships:

I am in constant touch with them all.

Pippa, MND interviewee

These relationships, built on empathy and shared experiences were very important to forum users and could lead to long-term friendships.

##### Friendship

Friendships operated at different levels, within groups or communities, or in one-to-one relationships. The majority of interviewees described their relationships with other forum users as friendships, sometimes qualifying the description, for example, as forum friends or cancer friends. Some interviewees were keen to emphasize that these were friendships in the truest sense (ie, these friendships were felt, emotional bonds). Some MND interviewees talked about feeling a closeness and affinity to their forum friends, bonded by the unique experience of MND. The majority of interviewees had never met these friends face-to-face and did not have plans to do so, but they supported one another within the online spaces. It was not uncommon for interviewees to remark about the paradox that these forum friends knew more about them than some their offline friends:

...I mean, I always remember one of the people on the forum just sort of saying who would have believed you could get so much support from a computer and a bunch of strangers? Which I think is an absolutely wonderful quote, um, and actually is very true...we are in a sense strangers but we also know each other better than you probably know most—a lot of people in your life really. It’s a very strange relationship.

Janice, breast cancer forum interviewee

Conversations were found to move away from the forum so that private thoughts could be expressed without fear of being seen by others:

There was another lady that, um—she was in a different group than I was at the time and we actually still email each other regularly now, um, and I suppose she’s the only person that I have really opened up to and likewise her to me. We’ve never met. She lives up north—um, she was a couple of months behind or one month behind and we used to talk and then we’d talk about our darkest moments and our fears and, you know, our families and things. So she’s—to me, it’s more of an intimate relationship.

Frances, breast cancer interviewee

Let’s take this thread to email and let’s keep in touch.

MND forum post

Friendships provided a space for empathy, where the connections and understanding enabled empathic feelings to grow:

Yeah, understanding somebody, empathy, all those terms you’d associate with a friendship...

Vincent, MND interviewee

...I feel for them when things are going bad and I’m glad for them when things are going well and I enjoy kind of talking to them.

Isobel, breast cancer interviewee

Friendship motivated users to act in empathic ways—watching out for one another and coming to the aid of friends in moments of need:

...we’re all going through similar experiences and I’ve made a number of...friends, err, who I would do whatever I can to assist...physically...or emotionally...

Vincent, MND interviewee

I lost my father in December and within the—put in a message—you know, I put oh, I’ve just had a phone call I didn’t want. You know, I’ve just—Dad’s just died. I had about 13 messages within an hour. So—you know, it’s—it’s been a huge support.

Christine, breast cancer interviewee

These were bonds of mutual support, whereby users gave and received comfort and support within the context of an ongoing relationship.

#### Connected by Feelings

Feelings were a strong theme within both forums, mentioned frequently both within interviews and forum posts. An emotional understanding of ill health formed an important means of connecting users. The expression of feelings and emotional vulnerabilities formed both a language of empathy and an empathetic cue for users to provide support to others in need. These remarkably honest and open expressions of feeling were encouraged by the anonymity of conversing within an online space. However, some interviewees were unaware that posts could be read by anyone searching the Internet and not all users felt comfortable with emotional expression on the forum. This view was expressed by a minority of interviewees reflecting their worries around emotions getting out of hand:

...I don’t personally discuss my deepest fears and how I feel on there...I think because it is opening the door again, you know, so once [unclear], you’ve got to deal with those emotions.

Frances, breast cancer interviewee

I just think the forum is for sharing experiences and really private matters about how you feel needs proper counseling otherwise it could get out of hand.

Robert, MND interviewee

Both forums had a strong emotional undercurrent because people within these spaces lived with fear and uncertainty, but the depth of distress was perhaps more evident from these comments from one person within the MND forum:

I worry about my family because I won’t be there for them.

I have to leave a room to spare people from my uncontrolled emotions.

I cried after the diagnosis, my wife held me like a small child.

I used to laugh and tell jokes, now I fade into the background so others don’t have to slow conversation down whilst I tap away on the wretched smartphone. People look with sympathy and I want to scream.

For MND users, although deterioration and ultimately death were inevitable, they also faced uncertainty in their near futures about the nature of their decline and the speed with which it would happen. Users were brutally reminded of the reality of their situation when other users died, and although members of the Breast Cancer Care community also lost their lives to the disease, for MND sufferers the nature of the condition meant that end-of-life issues were more imminently germane for them. The fact that these losses were documented within the forum and experienced by the users affected the empathetic tone of the community. Members were bonded by their grief, the knowledge of their own mortality, and the loss of the person they once were, as the disease stripped away previous normalities. This made for a profound emotional connection with others sharing the same fate:

There is a tremendous empathetic bond between the forumites. We share a life sentence. It cannot be more powerful than that

The feeling between us all on the forum has been strengthened through all these deaths. It is tangible.

Pippa, MND interviewee

Within the Breast Cancer Care forum, death tended to be discussed within particular spaces, such as the Living with Secondary Breast Cancer end-of-life board; this effectively shielded the wider forum from the experiences and meant that users who were at a different stage of the illness (eg, awaiting a diagnosis) did not encounter these difficult issues when they were not ready for them. Forum users could choose whether they wished to view these discussions or they could avoid them altogether. This effectively created spaces within spaces, in which users who were facing difficult situations could discuss these openly with others, while the wider forum membership were protected from these conversations.

Although participants felt sadness or grief on hearing about the deaths of other users, the experience also highlighted individual vulnerabilities:

Um, someone on the Younger Breast Cancer Network died last weekend and I have to say I did shed a tear, even though I’ve never met her, um, just because of the really sad story of it all. So um, I don’t know because it’s a bit mawkish sometimes to read—read the sadder things that happen but you have to be aware that it’s a possibility...

Libby, breast cancer interviewee

Users from both forums understood the emotional cost of living with ill health. The emotions and tensions of living with uncertainty provided a language for empathetic expression.

##### Expression of Feelings

Discussing feelings was a recurring theme within both health forums. Some expressions used to describe feelings were common in both forums, such as the emotional roller coaster many experienced at the time of diagnosis or during other points of particular turmoil. Feeling alone was also a commonly expressed emotion, as people sought to deal with the isolation of living with health conditions that separated them from others, both emotionally—and especially for people with MND—physically:

I imagined saying goodbye to my children, thought about the instructions and letters I would need to leave...Was wondering about at 3 am scared lonely frightened I am sure that most of us feel like this but isn’t it horrible!!

breast cancer forum post

My legs failed first...but I have found it harder now my arms are deteriorating badly. I don’t know if anyone here has ever felt like they’re alone but especially at my age it is hard to get my head round this day to day.

MND forum post

Emotional venting provided an important means of “letting go” of emotions, of catharsis, an expression more powerful and meaningful because it was undertaken with an audience of users going through the same thing. Users also asked others to validate feelings, often asking specifically if anyone else within the forum shared the same feeling. This request occurred more frequently within the Breast Cancer Care forum, but was also found within MND posts, as illustrated in the previous quotation. The need to check out emotions seemed to occur at times of great emotional intensity or when users experienced unexpected feelings:

All this is good news [now that I have got to the end of treatment] and yet why am i so fed up? People say how well I’ve done and now it’s nearly over, but I feel like crying all the time like I did in the beginning.

breast cancer forum post

These types of post often motivated forum members to reach out and to offer reassurance that the emotions described were normal and shared by others:

...you’ll find somebody [who can relate to the emotion] who can kind of pitch in say—validate that yes, you’re not going crazy. [laughing]. You’re just being like that today or whatever it is, it just kind of—it’s part of that roller coaster that you’re just going to have to go through...

Danielle, breast cancer interviewee

...Your [sic] not on your own feeling the way you do, I know I [expletive] well try very hard to hang onto keeping sane, so don’t think everything is down to you, it’s what this [expletive] disease does to you...I understand the loneliness you feel even with people around don’t help much, and seeing what we had now become what you have now...

MND forum post

Users shared their own emotional experiences with the intention of comforting others. Libby (a breast cancer interviewee) spoke of sharing in order to calm and to reassure, “...to calm her (another forum user) down and say ‘Well yes, totally understand what you’re going through, been there...’” People from both forums sent encouraging messages to struggling forum members either sending “strength” or advising that the users should “keep strong.”

##### Emotional Impacts

The emotional impact of serious illness was understood within the forum where all participants could relate to the impact of the illness on relationships and daily life. Participants described giving up jobs they loved and they discussed the pressures placed on families, as partners became carers, relationships were put under pressure, and friendships did not necessarily last. Some participants felt that there was not the same understanding of the emotional consequences of illness from people who had not experienced it first hand, but they were able to gain this from other forum users who had been through similar experiences:

um, you talk to somebody on this forum and he has a—perhaps a better emotional understanding of where you are, not just the physical stuff but, you know, he’s perhaps been through the emotional side and with great respect to the medic—the medics, they might have seen it but they haven’t done it.

Terry, MND interviewee

...you see, the thing is the medics are very good here, err, but there’s no emotional support. That is completely what’s missing so you know...

Christine, breast cancer interviewee

Members of both forums documented struggles to deal with the emotional impact of living with a changed body. The MND forum users reported physical losses as a result of disease progression, whereas users on the Breast Cancer Care forum posted experiences of coming to terms with a body changed as a result of therapies or surgery. They worked through the implications of this for social and personal identity as well as the ability to carry out everyday tasks. Some feelings were more likely to be expressed within one particular forum or space within the forum, for example, the monthly chemotherapy threads on the Breast Cancer Care forum. Frustrations regarding the physical realities of living with MND were commonly expressed, with users often describing the impact of the disease on their body and everyday activities. Part of this frustration was borne out of the “battles” that some users experienced to get appropriate support from services to manage their condition and also the lack of progress in finding a cure for MND:

...frustration is probably the only emotion I feel constantly; frustration, not anger.

Vincent, MND interviewee

For those living with breast cancer, fear was commonly expressed (eg, fear of not surviving, fears about test results, and emotional strain of living with such fears day to day). When articulated, these emotional outpourings had particular resonance within these spaces and elicited great empathy from others:

...I’m reading this because I’m in the same boat—anxious and fearful especially at night. I wish I had more peace of mind...

breast cancer forum

## Discussion

### Principal Findings

The aim of this paper is to develop a better understanding of how empathy develops and operates in online spaces where people share information, experiences, and emotions relating to living with a serious illness. Although other papers on empathy have tended to quantify and deconstruct processes of empathy documented on forums, our study provides a deeper understanding of the human experiences and human processes that build and foster empathy. Our approach of combining both interview and forum data enabled us to gain a new and deeper understanding of the processes of how empathy is developed and operates within the forums and therefore provides a novel contribution to current literature. The interview data enabled us to situate empathy building within the wider human experience of ill health and to hear directly from participants how they experienced empathy within the space, their thoughts, and actions. The forum data provided a demonstration of how these processes were enacted within the two forums. The analysis provides a unique insight into the development and operation of empathy from the perspective of participants with two very different health conditions. We found that although differences existed, there were points of similarity and key to this was the experience of uncertainty found in both conditions. These experiences both drew participants to the online forums and made for empathetic spaces. Our study found a common means by which empathy is built within both online health forums. Empathy emerges as a process beginning outside the forums in the shared experience of diagnosis, and then develops and operates within the forums through connections sought and made among users.

Participants experienced diagnosis as a life-changing event. This shocking and devastating event marked the transition from previous normalities, into an uncertain world [34-36]. The sense of devastation was particularly marked for participants with MND told that they had a debilitating condition without cure [29,36-38]. Participants with both health conditions described a period when they struggled to make sense of their situation, and felt the need for support and information [38-40]. Participants felt that there was insufficient focus on the emotional impacts of living with breast cancer and MND [41,42]. These intensely emotional experiences both drew participants to the forums and provided an emotional understanding which informed empathetic interactions with other users [15,43]. Other studies have observed that forum participation can be prompted by negative experiences within the offline world [44]. Interestingly, most participants in our study came across the forums while searching on the Internet [45], and although they may not have been consciously seeking empathetic support, they recognized the potential value when they came across it.

Both forums were experienced as very human spaces, connecting people to a community of shared experiences. The communities were *felt*, and were perceived as warm and comforting communities, where members worked to support one another within a shared supportive ethos [46,47]. This feeling came primarily from the sense that they were connecting to human experience [14,48]. The dimensions of experience discussed within both forums felt more meaningful and true to the lived experience than interactions with health professionals focusing on medical aspects [42] or with their family where conversation may be constrained by fear of causing upset [12]. It is the case that some users can feel distant from their usual sources of support, most frequently family and friends [49]. Often they do not want to burden them with their fears or they are reluctant to share because they feel that they will not adequately understand the experience [12,50,51]. The useful and relevant sharing undertaken within both sites provided a unique source of support, of experiential and practical information, and emotional support [6]. This fit between support provided by the forum and what participants felt they needed echoes the idea of empathetic accuracy [22].

The informational and emotional support provided within both forums fit within the criteria of social support [52,53]. However, the added element of empathy from others with greater homophily profoundly altered the experience of giving and receiving social support within the context of both online health forums. Consider, for example, emotional support. The fact that users from both forums shared first-hand experience of what it is like to live with both conditions took this emotional support to a higher level. Users knew from the inside what it meant to live with the differing conditions and what is at stake, and used this to inform empathetic responses. Emotional support within this context was considered more meaningful (eg, users were able to use short-hand descriptions to describe situations and they knew others within the forum would recognize and understand). The emotional support was highly valued and trusted [17], coming as it did from an audience of others sharing similar situations and who took the time to provide support even though they themselves may be suffering negative impacts of ill health. Emotional support provided by both online health forums differed from that provided by family, friends, and health professionals. Family and friends could imagine/approximate what the person with breast cancer or MND was going through, but the people within the forums knew. Health professionals provide support based on clinical expertise and observations of patient experiences, but again this is based on indirect experience. Thus, within the communities created by both online health forums, users found a unique source of social support with empathy that could not be found elsewhere. This paper provides a deeper understanding of the importance of social support with empathy provided by peers within online health forums.

Most participants in our study experienced both health forums as very human, intimate spaces, within which they felt empowered to share personal information and experiences. Both forums were understood as a community of people connected by the same traumatic experience, where human understanding and comfort emanated from shared experiences and from sharing their experiences with one another [1]. Sharing experiences, as a way to support others, is foundational in the process of creating empathy; users share personal experiences, information, and emotions in the hope that it can help others [19]. Empathy was encouraged by a willingness to express vulnerability and this was commonly found in posts shared on both forums. These open and honest narratives encouraged fellow users to reciprocate in kind, opening up spaces for empathy. Participants were encouraged to share by connections that they formed to other users, based on shared interests, and within relationships and via expression of shared emotions. Both forums provide a means of connecting with others at a one-to-one level or in subgroups with shared interests, and technologies enabled conversations to move in and out of the forum. Thus, empathy was built within different spaces, groups, and technologies, as users sought to work through the emotional and practical work of living with an uncertain future [54]. What was clear from our analyses was that sharing and making connections are dependent on each other: sharing facilitates the development of connections and making connections encourages sharing.

Within both spaces, users sought out connections based on similarity. Within the literature, similarity is considered a key facilitator of empathy (ie, people who are similar are more likely to empathize) [19] and to detect another person’s feelings with accuracy [22]. Interviewees gave accounts of connections made with people in similar circumstances or with shared experiences, which provided opportunities to interact on a profound level. The level of similarity sought varied between the two conditions [55], with participants with breast cancer more likely to seek out others sharing a specific diagnosis or undergoing similar treatment [51,56], reflecting the varied types of breast cancer and differing treatment pathways. In such instances, participants were better able to open up and disclose fears, perhaps because shared risks and illness identity created a stronger tie strength [57]. The fact that users actively created new spaces to share with similar others demonstrates the need for spaces which provide relevant support and an environment which encourages users to unburden their fears.

Empathy was fostered by relationships developed in the forums. Participants described how they sought out or chanced upon an individual or group that they felt either an affinity or a connection to, because of a shared similarity. Relationships grew and deepened through acts of narrative sharing. Initial phases of sharing may have focused on the illness experience, but soon broadened to encompass everyday experiences [1,30]. Over time, users gained an understanding and a sense of the other person, their personality, circumstances, and illness experiences [14,19]. These acts of sharing and connection enabled feelings to grow for fellow users [30], and encouraged empathetic and supportive behaviors [15,19]. Friendships provided an emotional space for users to express their deepest fears in acts of unburdening [25,30]. Online friendships provided a buffer against the isolating impacts of ill health [35]; this was of particular importance for participants with MND, for whom speech and mobility difficulties may combine to inhibit interactions in the offline world [2]. Conversations between friends were shared in the open forum, including within the spaces dedicated for specific groups, such as the monthly chemotherapy threads and the board for lesbian and bisexual women on the Breast Cancer Care forum, as well as in external spaces such as closed Facebook groups where privacy may foster even greater intimacy [58]. Although there is a risk that conversations within these private spaces might take valuable sharing away from the health forum [59], these alternative spaces were needed and supported practices in everyday life whereby people converse both within groups and more openly within private conversations.

Emotions were an important facilitator of empathy. The experience of ill health was “felt” by interviewees who described in vivid terms the emotional impacts of breast cancer and MND. This understanding of what is *feels* like to live through a journey from diagnosis into the unknown, and the wider impacts on family and previous ways of living, was shared, recognized, and understood within the forums [3,14]. Participants perceived that they gained an emotional understanding from the forums that was not found elsewhere because it came from both *lived* and *felt* experiences [60]. Feelings were anchored in experiences, thus enabling users to recall how they felt at particular times within their illness [30,61]. This motivated users to reach out and support others, and provide the type of support that was needed, particularly during times of great emotional need [19]. This recognition of feelings by others within both forums was a key element bonding users together into an empathetic community [60].

The study demonstrates the importance of time in the development of empathy. Participants from both health conditions described individual journeys, going from diagnosis, to finding the forums, and building connections and friendships within the forums. These processes occurring over time facilitated the development of empathy. Users took time to get to know one another. Empathy developed and grew within relationships. Thus, time both underpinned the development of empathy and intersected with the key enablers of empathy identified by this study. This interconnection between temporality and other key enablers is noted by a study exploring the development of trust in online health forums [17].

Empathy was built within both forums on the same building blocks of shared experiences and connections; however, the breast cancer and MND forums also differed in some respects. The differing disease trajectories altered how empathy was enacted within the two forums. The hoped-for trajectory in breast cancer of treatment and recovery was played out within the forum, with waves of new users joining the monthly treatment groups and working their way through varied treatment pathways. The emotional peaks and troughs of these individual journeys, and the fact that the majority of the users were female [23], all shaped how empathy played out within the space. The MND forum had fewer members and fewer boards within the forums, reflecting the rarity of the condition, the lack of a curative pathway, and the focus on supportive treatments and living with and managing the condition. Users were bonded by shared experiences; the debilitating impact of the conditions stripped away independence and narrowed interactions with the outside world. This made for a distinctive type of empathetic bond [2]. The gender balance of the forum also differed. The majority of core members, posting most frequently, were male; however, the site itself was mixed gender [62]. There was also a greater presence of family caregivers on the site than that found on the Breast Cancer Care forum. However, despite these differences there were clear points of similarity, with participants from both conditions experiencing uncertainty. For participants with breast cancer, this often meant an ongoing fear of not knowing what would happen to them in the future (eg, treatment outcomes or risk of secondary cancers). Participants with MND knew that they would deteriorate, but did not know when and how fast this would happen, and how it would impact on their lives. People from both conditions dealt with this by coming together on forums to make sense of their situation within the empathetic space provided by the forums.

### Strengths and Limitations

Our findings are strengthened by combining interview data and forum posts, the two sources providing differing views and perspectives [16,63] as well as new insights. Use of both datasets together strengthened our analysis and the validity of our claims [17]. This approach has particular value when researching a topic such as empathy, which is abstract and intangible [64]. We combined these differing methodological approaches with the exploration of two contrasting health forums and argue that this approach yielded a richer level of analysis and deeper understanding [63].

This paper adds to knowledge of how empathy is defined by users of health forums. The definition utilized was chosen because it matched interviewee participant definitions. Core elements of this definition—knowing and feeling—could be defined as emotional intelligence, informed by experiences specific to the health conditions. Users knew what others in the forums were feeling, recalling (often vividly) how they themselves had felt during the stage of illness identified or feeling a resonance with emotions described. Users were able to imagine themselves in the circumstances of others, using this understanding to inform compassionate and considered responses.

Interviewees were self-selecting and there is a danger that they represented an overly positive view of health forums, although participants did discuss both positive and negative issues. The interview data were combined with forum data, which provided insights into the perceptions and experiences of a wider body of forum members [65]. However, neither of these approaches can represent the views and experiences of individuals who choose not to share in online environments (eg, people who lurk and read posts but do not post themselves or who are digitally excluded). A further limitation is that there were fewer interviewees with MND, possibly reflecting perhaps the physical and communicative barriers to participation in research for people with this condition.

### Implications for Practice

The study highlights areas of unmet need for individuals living with breast cancer and MND, particularly around emotional and informational support [37,38,66]. Forums provide a means of fulfilling needs within a supportive peer-to-peer environment, and that aspect is particularly valued [65]. Study participants commonly found the forums while searching the Internet, rather than being signposted by health or social care professionals. The potential value of forums to patients and families may not be fully understood by health professionals [44,45], and additional factors, such as level of awareness and time constraints, may reduce the likelihood that professionals will sign-post patients to forums. Raising awareness of the potential benefits of online health forums among health professionals could help them to encourage patients to use these as support, to supplement the care that they, as health professionals, provide.

The study highlights important issues for organizations hosting online health forums. Our research brings a new understanding of how users operate within the forums, seeking support that they perceive as beneficial to fulfill emotional and practical needs. Hosts may consider how best to enhance forums to foster elements that users found most helpful and appealing. The human aspects were of key importance (ie, the supportive and warm atmosphere, the ability to connect with others who shared and understood, and to build relationships). Provision of useful information on practical support was also a key element. Thus, structures or affordances are needed to enable users to both find useful information and satisfy needs to connect with others [47]. This requires empathetic design, which should reflect the needs of users coming to the space, but also provides a means of enabling users to interact in an empathetic way within the space [67]. Design needs to take account of needs changing over time [50], for example, so that there are spaces available for different stages of an illness. Attention should also be focused on protecting the supportive atmosphere of the space [46] because this was perceived to be more conducive to empathetic interactions. Forum moderators might consider how best to moderate spaces to protect the supportive and warm ambiance.

Users interacted with one another in different spaces and conversed in different ways within the spaces, often sharing most private fears within a one-to-one setting. Thus forums should reflect these differing needs, providing access to differing levels of communication (forum, subgroups, one-to-one messaging). Some users may leave the forum space to form special interest groups within other platforms (eg, Facebook). Forum hosts may consider how best to interact with Facebook groups, given the popularity of this social media site [68] and the ease with which social media enables people to form groups independently. Health forums exist within this dynamic space, and thus it should not be considered a failure if users meet within the forum and move off into another setting. The informal, less-anonymous space provided by Facebook may enable users to get to know one another with greater immediacy, and thus encourage empathy. However, there are risks associated with interacting within a private unmoderated space [69] of which users should be aware.

### Implications for Future Research

Further research on empathy in online health forums could examine barriers to the development of empathy and how these affect people’s sharing of their information and experiences, as well as how they receive information from people with whom they have little or no empathic connection. Although our research has highlighted the importance of sharing in the development of empathy, and how empathy can lead to further sharing, there was little evidence from the discussion boards we sampled, or from the interviews, of how conflicts and disagreements within the forums affect empathy and sharing, or of the extent to which empathy acted as a buffer during disagreements and enabled people to continue to share experiences and emotions. Further research could also examine how empathy operates in relation to other aspects of the lives of people with life-threatening or terminal illnesses in relation to sharing information and experiences; for example, perceptions of the risks of sharing and self-disclosure, and the importance of trust [17] in the development of empathy. As new online platforms and functionalities develop, and people become more aware of issues relating to privacy, developing a better understanding of how empathy and trust operate will enable forum designers and providers to develop spaces in which users feel confident about sharing information, experiences, and emotions.

### Conclusion

This study contributes new knowledge to the underresearched and important area of how empathy develops and operates in online environments by an exploration of two online health forums. Our study demonstrated that empathy develops through shared experiences and connections. The process begins outside the forums with the transformative experience of diagnosis. Forum users in our study were motivated to seek online support to meet emotional and informational needs unfulfilled by usual sources of support. They were often at a point of great emotional need, ready to both receive and give empathy. These empathic processes are developed through connections formed on the basis of shared needs, feelings, similarities, and relationships. Empathy was also fostered by a range of structural possibilities within the forums which gave users the opportunity and means to interact within public, restricted, and more private spaces, as well as within groups and one-to-one exchanges. The atmosphere and feeling of sites and perceived audiences were also important, with forum users sharing a perception of a virtual community of caring and supportive people, offering comfort and support. Our findings are of value to organizations hosting health forums that seek to offer support to individuals living with long-term and acute life-threatening conditions. Our findings show the importance of providing and protecting how empathetic interactions are formed and maintained, given the key importance of these interactions in encouraging sharing among forum users, which in turn nurtures well-being and resilience.

## Abbreviations

MND: motor neuron disease
